# Erratum: Plant to animal protein ratio in the diet: nutrient adequacy, long-term health and environmental pressure

**DOI:** 10.3389/fnut.2023.1281700

**Published:** 2023-09-01

**Authors:** Frontiers Production Office

**Affiliations:** Frontiers Media SA, Lausanne, Switzerland

**Keywords:** healthy dietary patterns, nutrient adequacy, environmental footprints, diet optimization, plant-based diets

Due to a production error, the reference for “46” was incorrectly written as “Mariotti F, Huneau JF, Fouillet H. No nutritional lessons can be learned from a misspecified and overrestricted model with no sensitivity analysis. *J Nutr*. 137:1383–9. doi: 10.1093/jn/137.6.1383”.

It should be “Mariotti F, Huneau JF, Fouillet H. No nutritional lessons can be learned from a misspecified and overrestricted model with no sensitivity analysis. *J Nutr*. (2023) 153:911–2. doi: 10.1016/j.tjnut.2022.12.021.

The publisher apologizes for this mistake. The original article has been updated.

